# Surgery first - prediction of surgical movements based on untreated cases

**DOI:** 10.1186/1746-160X-10-S1-O3

**Published:** 2014-12-12

**Authors:** Thomas Stamm, Susanne Jung, Gudrun Prigge, Johannes Kleinheinz

**Affiliations:** 1 Department of Orthodontics, University Clinic Münster, Münster, Germany; 2 Department of Oral and Maxillofacial Surgery, University Clinic Münster, Münster, Germany

## Aim

The aim of the study was to test the following hypothesis: There is no difference in orthognathic surgery protocols planned on pre-treatment patient records (Surgery First protocols) compared to surgery protocols planned on patient records after orthodontic preparation (Surgery Late protocols).

## Material and methods

Study type: Prospective, randomised, semi blinded trial. One hundred fifteen traditional treated orthognathic surgery cases were included based on inclusion and exclusion criteria. Pre-treatment records of the 115 cases were presented to a surgery team (surgeons, orthodontists) to generate a Surgery First protocol. The Surgery First protocols were then compared with the true (Surgery Late) protocols of the treated cases. The statistical power was 95%.

## Results

Surgery First and Surgery Late protocols of the same cases differ significantly. Impaction of the maxilla is the most predictable surgical movement with a mean error radius of 2.4 mm ± 1.9 mm for each measurement landmark. Maxillary advancement showed an error radius of 3.2 mm ± 2.9 mm. The highest errors were found in mandibular advancement / set back with 6.3 mm ± 4.3 mm on each side. Overall the errors add up to 14.8 mm ± 6.8 mm. Angle classes do not differ significantly concerning the planning errors.

## Discussion and conclusion

The hypothesis that there is no difference between Surgery First and Surgery Late protocols must be rejected. Planning errors with the Surgery First concept are not predictable. The post-operative malocclusion generated with Surgery First could lead to situations which are not manageable with orthodontics. There is a high risk of unfavourable occlusion and further corrective surgery at the end of treatment. The initial Angle class is not a valid predictor of low or high planning errors.

